# What does absence of lymph node in resected specimen mean after neoadjuvant chemoradiation for rectal cancer

**DOI:** 10.1186/1748-717X-8-202

**Published:** 2013-08-19

**Authors:** Won-Suk Lee, Seok Ho Lee, Jeong-Heum Baek, Woon Kee Lee, Jung Nam Lee, Na Rae Kim, Yeon Ho Park

**Affiliations:** 1 Department of Surgery, Gil Medical Center, School of Medicine, Gachon University, 1198 Guwol-dong, Namdong-gu, Incheon 405-760, Korea; 2 Department of Radiation Oncology Gil Medical Center, School of Medicine, Gachon University, 1198 Guwol-dong, Namdong-gu, Incheon 405-760, Korea; 3 Department of Pathology, Gil Medical Center, School of Medicine, Gachon University, 1198 Guwol-dong, Namdong-gu, Incheon 405-760, Korea

**Keywords:** Rectal neoplasm, Surgery, Neoadjuvant chemoradiation

## Abstract

**Background:**

The effect of insufficient node sampling in patients with rectal cancer managed by neoadjuvant chemoradiation followed by surgery has not been clearly determined. We evalulated the impact of insufficient sampling or even abscence of lymph nodes in the specimen on survival in patients at high-risk (T3, T4 or node positive) for rectal cancer.

**Methods:**

We conducted a single institution, retrospective analysis of all patients who underwent surgical rectal resection following neoadjuvant chemoradiation for treatment of mid to lower rectal cancer between 1997 and 2009. ypNX was defined as the absence of lymph nodes retrieved in the resected specimen.

**Results:**

A total of 132 patients underwent resection for treatment of rectal cancer following neoadjuvant chemoradiation. Ninety four patients (71.2%) were considered as having node-negative disease, including ypNx and ypN0. In 38 patients (28.8%), the primary tumor was associated with regional lymph node metastases (ypNpos). The mean number of retrieved nodes per specimen was 14.2, respectively. The five-year overall survival from initial operation for the ypNx group was 100%, respectively. The estimated five-year overall survival for ypN0 and ypNpos was 84.0% and 60.3%, respectively (*P* =0.001). No significant differences in overall survival were observed between the ypNx and ypN0 group (*P* =0.302).

**Conclusion:**

Absence of recovered LN in resected specimens after neoadjuvant chemoradiation was observed in 7.6% of specimens. Absence of LN should not be regarded as a risk factor for poor survival or as a sign of less radical surgery.

## Introduction

It is estimated that approximately 50 ~ 60% of rectal cancers are considered to be locally advanced tumors with clinical stage T3 or T4 or node positive disease, characterized by poor prognosis due to increased incidence of systemic and local recurrence and decreased long-term survival [1]. The preferred strategy for management of locally advanced mid to distal rectal cancer is multimodality treatment that includes radical surgery, chemotherapy, and radiation therapy. Radical surgery should be performed according to established surgical principles, which include en bloc resection of the tumor-bearing rectum and the adjacent lymphovascular pedicle, commonly referred to as total mesorectal excision [2].

The lymph node (LN) status remains one of the independant prognostic factors in rectal cancer. Patients without LN metastses have significantly better survival, compared to node positive patients, in the abscence of distant metastasis. However, a mininum number of LNs retrieved from the resected specimen is prerequisite to ensuring both adequate nodal sampling and surgical radicality [3]. Several studies have demonstrated a significant survival benefit of patients with node negative disease with an increased number of recovered nodes [4-6]. Chemoradiation exerts effects not only on tumor down staging and rate of metastatic LN but also on the overall number of retrieved nodes [7,8]. The number of LNs assessed pathologically is a combination of the aggressiveness of the surgeon in resecting widely around the primary tumor and of the didicated pathologist in searching the specimen for additional nodes. Inadequate retrieval of LNs is considered unacceptable for patients who have not undergone pretreatment and went straight to surgery. However, the effect of this finding in patients with rectal cancer managed by neoadjuvant chemoradiation followed by surgery has not yet been determined.

In an attempt to determine the impact of insufficient sampling (i.e. ypN ≤ 12) or even abscence of lymph nodes in the specimen (i.e. ypNx) on survival in these patients, we evaluated a cohort of patients who enrolled in our neoadjuvant chemoradiation protocol for patients with high-risk (T3, T4 or node positive) rectal cancer.

## Patients and methods

### Eligibility

Between September 1997 and September 2009, 720 patients with rectal cancer underwent treatment at Gachon Medical Center. The inclusion criteria for the study were as follows: 1) lesion located no more than 10 cm from the anal verge; 2) clinical TNM stage II and III (T2-T4, or N positive and M0) on abdominopelvic computated tomography; 3) patients with histologically proven rectal carcinoma; 4) age ≥18 years; 5) patients who underwent preoperative chemoradiation; 6) mid (anal verge 6 cm to 10 cm) and lower (anal verge 1 cm to 5 cm) rectal cancer patients. Of the 720 patients screened for rectal resection, 132 patients fulfilled the inclusion criteria and were included in this retrospective analysis.

### Pretreatment staging

Initial staging included complete physical examination, digital rectal examination, colonoscopy, serum CEA (carcino-embryonic antigen) abdominal and pelvic spiral CT scans, and endorectal ultrasonography or rectal magnetic resonance imaging in selected patient chest X-ray.

### Treatment

All patients received two initial cycles of chemotherapy followed by pelvic radiation therapy plus chemotherapy. The concurrent chemotherapy was performed at the first and fifth week of radiation with bolus intravenous 5-fluorouracil 400 mg/m^2^ and leucovorin 20 mg/m^2^ for five days per week. All patients received external beam radiation therapy (median dose, 50.40 Gy; range 48.4 to 55.8 Gy), according the previously published techniques [9,10]. Using 6 to 10 Mv photons, a 3- or 4-field technique was used.

Surgery was attempted at 6–8 weeks after completion of neoadjuvant chemoradiotherapy. All patients underwent low anterior resection or abdominoperineal resection and total mesorectal excision (TME) according to the surgical technique described by Heald et al. [11] as well as high ligation inferior mesenteric artery and en bloc resection of any suspected adjacent organ invasion. Four cycles of postoperative adjuvant chemotherapy with 5-fluorouracil 500 mg/m^2^ for five days was added.

### Staging

Two pathologists performed meticulous dissection and retrieval of mesorectal lymph nodes. A rigorous search of the mesorectum was performed in order to identify as many lymph nodes as possible. Each lymph node was analyzed in its entirety in separate blocks. When fewer than 12 lymph nodes were found, an additional 24-hour surfixation in Bouin’s fluid was performed in order to facilitate recovery of residual lymph nodes. No clearing technique was performed. Patients were staged according to the American Joint Committee on Cancer recommendations [12]. ypNX was defined as the absence of lymph nodes recovered in the resected specimen.

### Surveillance

Surveillance for recurrence following surgery was outlined as follows: physical examination, serum CEA, chest X-ray, and spiral abdominal CT scan were performed every six months for three years, and annually thereafter.

### Statistical methods

The primary endpoint of the study was overall survival. Overall survival (OS) was estimated using the Kaplan-Meier method. OS was measured from the date of diagnosis to the date of death or the last follow-up visit. Survival rates were compared for statistical differences using log-rank analysis. Chi squared and ANOVA were used for categorical and numeral variables between groups. Multivariate analysis was performed using stepwise Cox proportional hazards regression modeling. *P* values less than .05 were considered statistically significant and all *P* values correspond to two-sided significance tests.

## Results

### Patient characteristics

There were 95 men and 37 women enrolled in the study. The median age of subjects was 59 years (39-77). The median follow up from primary surgery was 54.2 months (range, 12.1-128.5 months). Primary lesions were found above 6 cm or more from the anal verge in 82 patients (62.1%) and below 5 cm in 50 patients (37.9%) (Table 1).

**Table 1:** Patient characteristics (n = 132)

**Variables**	**Number of patients**	**%**
Median age	59.0(39-77)	
≤ 60	61	46.2
> 60	71	53.8
Sex: M/F	95:37	
Primary tumor location		
Mid	109	47.0
Lower	23	53.0
Pretreatment CEA, ng/mL*		
Median	5.32(0.1-83.3)	
≤ 5.0	112	84.8
> 5.0	20	15.2
ypT stage		
Tx	9	6.8
T1	1	0.8
T2	29	22.0
T3	92	69.7
T4	1	0.8
ypN stage		
x	11	8.3
0	83	62.9
positive	38	28.8
Retrieved nodes		
Mean	14.2(0-31)	
Lymphovascular invasion		
Positive	30	77.3
Negative	102	22.7
Perineural invasion		
Positive	13	9.8
Negative	119	90.2
Cell differentiation		
Well	12	9.1
Moderately	104	78.8
Poorly or mucinous	16	12.1
Body mass index		
≤ 22	39	29.5
> 22	93	70.5

*CEA, carcinoembryonic antigen (normal range 0-7 ng/mL).

Numbers in parenthesis indicate ranges.

The median interval time between completion of preoperative chemoradiation and surgery was 6.2 weeks (range, 4.4-8.6 weeks). Among the 132 patients, 52 (39.4%) underwent low anterior resections with or without protective ileostomy; 39 (29.5%) underwent ultralow low anterior resections with or without protective ileostomy; 22 (16.7%) underwent Hartmann’s procedure and 19 patients (14.3%) underwent abdominoperineal resections. A summary of patient characteristics is shown in Table 1. In this study, 92 patients (69.7%) underwent a sphincter saving operation without permanent colostomy.

A total of 94 patients (71.2%) were considered as having node-negative disease, including ypNx and ypN0. In 38 patients (28.8%), the primary tumor was associated with regional lymph node metastases (ypNpos). The mean number of retrieved nodes per specimen was 14.2, respectively. The rate of complete sterilization, i.e., staged ypTxN0 and ypTxNx, was 6.8% (nine patients). Of the 130 patients with clinical stage III, 92 patients (69.7%) (9 + 2 + 28 + 53 patients) were downstaged after chemoradiation (Table 2). All of the ypNx patients (n = 11) had clinically significant lymph node enlargement(s).

**Table 2:** TNM downstaging in a series of 132 rectal cancer patients treated with preoperative chemoradiotherapy

	**CR**	**0**	**I**	**II**	**III**	**Total**
cII*	0	0	1	1	0	2
cIII*	9	2	28	53	38	130
Total	9	2	29	54	38	132

*Clinical stage II or stage III.

### Lymph node status

Pathologist A evaluated 74 patients and retrieved an average of 13 (range, 0-24) lymph nodes and Pathologist B evaluated 58 patients and retrieved 15 (range, 0-31) lymph nodes, respectively. There were 11 patients (8.3%) with absence of lymph nodes recovered in the resected specimen, i.e., ypNx (0 out of 0). The overall pathologic characteristics of ypT stage and ypN stage are shown in Table 3. In comparison of patients with ypNx and those with ypN0, no significant differences were observed in terms of gender, age, CEA, cell type, and body mass index. However, significantly lower risk of lymphovascular invasion was observed in the ypNx group, compared with the ypNpos group (*P =* 0.001) (Table 4).

**Table 3:** Correlation of ypT stage and ypN stage (n = 132)

**ypT stage**	**yp *Nx(% *)**	**yp*N0(%)***	**yp*Npos(%)***	**Total**
ypTx	3(2.3)	6(4.5)	0(0.0)	9(6.8)
ypT1	0(0.0)	1(0.8)	0(0.0)	1(0.8)
ypT2	4(3.0)	23(17.4)	2(1.5)	29(21.9)
ypT3	4(3.0)	52(39.4)	36(27.3)	92(69.7)
ypT4	0(0.0)	1(0.8)	0(0.0)	1(0.8)
Total	11(8.3)	83(62.9)	38(28.8)	132(100.0)

**Table 4:** Clinicopathological characteristics of patients according to ypN status (n = 132)

**Parameter**	***ypNx,%***	***ypN0,%***	***ypNpos,%***	***P*-value**	***P*-value, *ypNx vs ypN0***
Number of patients	11(8.3)	83(62.9)	38(28.8)		
Mean age(yr) ± STD	66.9 ± 9.13	59.5 ± 8.80	58.5 ± 11.4	0.434	0.725
Primary tumor location				0.514	0.680
Mid	11(100.0)	70(84.3)	28(73.7)		
Lower	0(0.0)	13(15.7)	10(26.3)		
Pretreatment CEA(ng/mL)	5.59 ± 5.98	4.02 ± 5.67	7.36 ± 17.73	0.229	0.926
Cell differentiation				0.765	0.443
Well	0(0.0)	7(8.4)	5(13.2)		
Moderate	8(72.7)	69(83.1)	27(71.1)		
Poorly	3(27.3)	7(8.5)	6(15.7)		
Perineural invasion	3(27.3)	3(3.6)	7(18.4)	0.102	0.288
Lymphovascular invasion	3(27.3)	9(10.8)	18(47.4)	0.001	0.435
Body mass index	22.18 ± 1.57	23.39 ± 3.14	22.86 ± 2.61	0.723	0.091
Retrieved nodes	-	13.45 ± 7.59	17.9 ± 6.87	0.720	0.047

*CEA* Carcinoembryonic antigen, *STD* Standard deviation.

### Risk factor analyses

In univariate analyses, CEA greater than 5, primary node status, and lymphovascular invasion had a statistically significant adverse influence on survival (Table 5). Although the results of multivariate analysis are limited by the small number of patients, results of stepwise Cox multivariate regression analysis revealed lymph node metastasis as a single independent prognostic factor affecting overall survival (*P* = 0.029; relative risk, 2.233; 95% Confidence Interval = 1.087-4.587).

**Table 5:** Univariate predictors of adverse outcome (n = 132)

**Variables(N)**	**5-year survival,%**	***P*-value**
Age		0.053
≤ 60	85.9	
> 60	77.0	
Primary tumor location		0.004
Mid	80.1	
Lower	70.7	
CEA, ng/mL*		0.001
≤ 5.0	84.3	
> 5.0	45.2	
Pathologic N stage		0.003
yNx	100	
yN0	85.0	
yNpos	60.3	
Lymphovascular invasion		0.642
Positive	70.8	
Negative	78.8	
Perineural invasion		0.951
Positive	75.0	
Negative	79.7	
Cell differentiation		0.709
Well	75.0	
Moderately poorly	79.7	
Body mass index		0.838
≤ 22	73.4	
> 22	77.1	

*CEA* Carcinoembryonic antigen (normal range 0-7 ng/mL), *N* Number.

### Disease recurrence and survival

**Figure 1:**
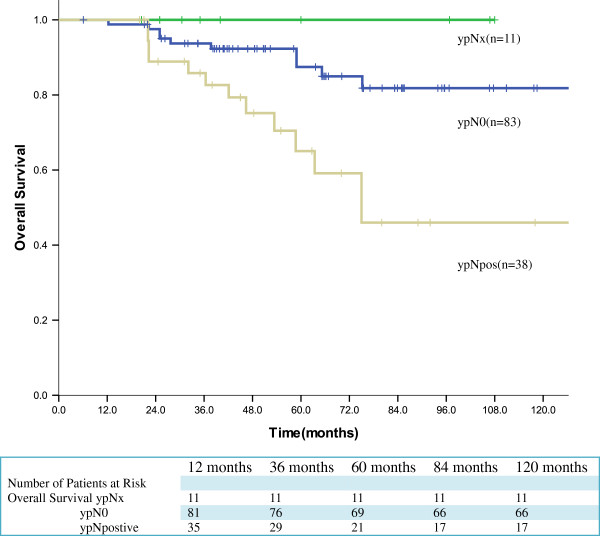
Overall survival according to according to ypN status (n = 132) (p = 0.003).

**Figure 2:**
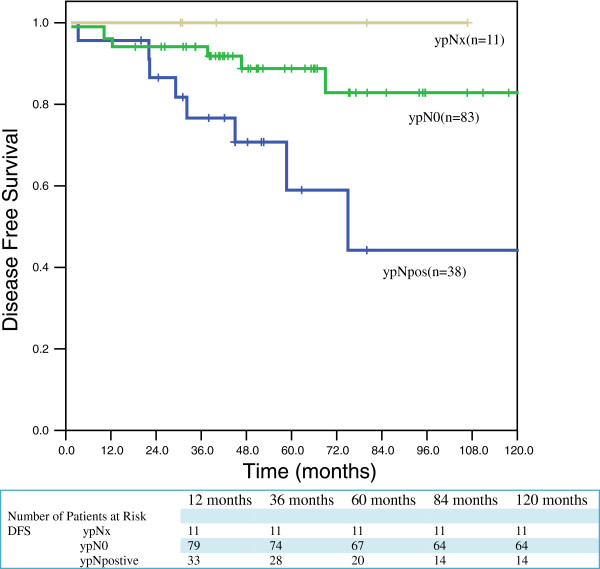
Disease free survival (DFS) according to ypNx status (n = 132) (p = 0.022).

The overall recurrence rate was 18.3% (25 patients) and overall cancer-related mortality was 17.4% (23 patients). No recurrences were observed among the ypNx group. Comparison of recurrences and cancer-related deaths showed significant differences between the ypNx and ypN0 group (*P* =0.032) (Table 6). Five-year overall survival for patients with ypNx group 100%. The estimated five-year overall survival for ypN0 and ypNpos was 84.0% and 60.3%, respectively (Figure 1). The estimated five-year disease free survival for ypN0 and ypNpos was 82.8% and 58.9%, respectively (Figure 2). No significant difference in overall survival was observed between the ypNx and ypN0 group (*P =* 0.302). However, the ypNpos group showed significantly worse five-year survival than the ypNx and ypN0 groups (*P =* 0.002).

**Table 6:** Recurrence according to ypN status (n = 132)

**Parameter**	***ypNx***	***ypN0***	***ypNpos***	***P*-value**
Recurrence	0	11	14	0.032
Cancer related mortality	0	10	13	0.002

## Discussion

Neoadjuvant chemotherapy and radiation therapy for treatment of locally advanced rectal cancer is a widely accepted treatment before surgical operation. Although it was initially used to improve rates of sphincter preservation and to optimize patient tolerance, the ideal number of nodes for rectal cancer surgery has been an issue of controversy. Many studies have demonstrated that the number of lymph nodes involved with a tumor has a strong impact on outcome for patients treated for rectal cancer [3]. Indeed, the TNM staging system is based on whether one to three nodes are involved or four or more [12]. An apparent increase in the number of retrieved nodes in patients with N1 or N2 disease, when compared to N0 disease, has been reported [3,6]. Although there is no clear agreement on the absolute number of total retrieved nodes, [3,13-15] the American Joint Committee on Cancer (AJCC) has recommended at least 12 lymph nodes as the standard for adequate staging of colon and rectal disease [12,16]. The AJCC staging does not include an exception criteria for pretreated rectal cancer, which in fact may have several factors that interfere with lymph node retrieval after rectal cancer surgery. In addition, the impact of absence of lymph nodes in the resected specimen after radical surgery for treatment of mid to distal rectal cancer after neoadjuvant chemoradiation has not been clearly defined in terms of overall survival.

In this study, the mean number of lymph nodes recovered after neoadjuvant chemoradiation followed by radical surgery was 14.2 LN per specimen, whereas the rate of ypNx was 7.6%. Findings of this series showed a statistically significant higher number of nodes in node positive patients, compared with node negative patients. For example, Wong et al. [17] reported that a mean of 14 nodes was found in node negative patients, as compared with 20 nodes in node positive patients. Unlike the study reported by Gorog et al. [18] and Kuo et al. [19], in this study, body surface area did not affect the number of LN retrieved. The major weakness of this study is that the results are based on 11 patients with no recurrent disease after ypNx. The retrospective nature and small sample size might actually have affected statistical analysis.

In a population-based study, including 5000 patients with rectal cancer from the SEER (Surveillance, Epidemiology, and End Results) database, results of multivariate analysis showed that patients who underwent preoperative radiation had significantly fewer recovered nodes, as compared with patients treated by adjuvant therapy [20]. The main reason for more retrieved nodes in this study may have resulted from complete total mesorectal excision with high ligation of inferior mesenteric vessels; however, a more likely explanation is the pathologist’s determination to retrieve as many nodes as possible.

The clinical impact of decreased retrieval of LN after neoadjuvant chemoradiation has not yet been clearly defined. It is generally believed that examining a greater number of nodes increases the likelihood of proper staging and thus might benefit from adjuvant therapy. Is survival in the ypNx population worse than that in the ypN0 population? In our series, the answer is no. The ypNx group exhibited a tendency toward better overall survival than the ypN0 group. The ypNx group may reflect an increased sensitivity to chemoradiation, which ultimately results in downstaging, as suggested by Habr-Gama et al. [5]. In fact, some of patients with ypNx could have been node positive patients before neoadjuvant chemoradiation. The results of the current series should be interpreted with caution. Due to the retrospective nature of this study, the analysis of disease free survival, local recurrence free survival and overall survival lack significant variables having a major impact on the outcome. Based on our results, the authors of this study suggest that the current recommendation of minimum requirement for LN retrieved (i.e. more than 12 nodes) for proper staging in these subsets of patients may be inappropriate and that conduct of more larger studies comparing the therapeutic outcome of ypNx and ypN0 is definitely warranted.

A wide range of tumor responses after preoperative chemoradiation therapy have been reported [2,6,9,21,22]. The reason for this wide variability in tumor responses is unclear. The results of studies reporting predictive clinicopathologic factors of tumor response are controversial and patients in the ypNx group may be associated with increased sensitivity toward chemoradiation therapy, and, thus, toward better survival. Some molecular biomarkers and various enzymes that may predict tumor response to chemotherapy have been suggested [19,23-25]. Neoadjuvant chemoradiation therapy has played a critical role in improving resectability and downstaging tumors. A variety of neoadjuvant chemoradiation regimens and radiosentisizers should be investigated for improvement of tumor response after chemoradiation and further prediction of tumor response.

In conclusion, absence of recovered LN in a resected specimen after neoadjuvant chemoradiation is rare and was observed in 7.6% in this series. Patients with ypNx after neoadjuvant chemoradiation and radical surgery may not be considered as patients at high risk for development of recurrence.

## Competing interests

The authors declare that they have no competing interests.

## Authors’ contributions

WSL provided the original idea and drafted the manuscript. SHL contributed in manuscript preparation. JHB contributed in the statistical analysis. WKL and JNL contributed in data collection and analysis. NRK contributed in pathologic reading of the data. YHP drafted the manuscript. WSL provided the concept and design of the study, WKL, SHL and JHB is responsible for data collection and analysis, WSL, YHP and NRK participated in the sequence alignment and drafted the manuscript. All authors read and approved the final manuscript.
